# Transcriptional Profiling of Protein Expression Related Genes of *Pichia pastoris* under Simulated Microgravity

**DOI:** 10.1371/journal.pone.0026613

**Published:** 2011-11-02

**Authors:** Feng Qi, Chao Wang, Yanli Liu, Imdad Kaleem, Qian Li, Chun Li

**Affiliations:** 1 School of Life Science, Beijing Institute of Technology, Beijing, Peoples Republic of China; 2 School of Chemistry and Chemical Engineering, Shihezi University, Shihezi, Peoples Republic of China; 3 College of Biotechnology, Tianjin University of Science and Technology, Tianjin, Peoples Republic of China; University of South Florida College of Medicine, United States of America

## Abstract

The physiological responses and transcription profiling of *Pichia pastoris* GS115 to simulated microgravity (SMG) were substantially changed compared with normal gravity (NG) control. We previously reported that the recombinant *P. pastoris* grew faster under SMG than NG during methanol induction phase and the efficiencies of recombinant enzyme production and secretion were enhanced under SMG, which was considered as the consequence of changed transcriptional levels of some key genes. In this work, transcriptiome profiling of *P. pastoris* cultured under SMG and NG conditions at exponential and stationary phases were determined using next-generation sequencing (NGS) technologies. Four categories of 141 genes function as methanol utilization, protein chaperone, RNA polymerase and protein transportation or secretion classified according to Gene Ontology (GO) were chosen to be analyzed on the basis of NGS results. And 80 significantly changed genes were weighted and estimated by Cluster 3.0. It was found that most genes of methanol metabolism (85% of 20 genes) and protein transportation or secretion (82.2% of 45 genes) were significantly up-regulated under SMG. Furthermore the quantity and fold change of up-regulated genes in exponential phase of each category were higher than those of stationary phase. The results indicate that the up-regulated genes of methanol metabolism and protein transportation or secretion mainly contribute to enhanced production and secretion of the recombinant protein under SMG.

## Introduction

Microgravity has significant effects on numerous microbial characteristics [1]. And studies concerning the influences of microgravity on microbial cells are drawing much attention because such information will lead to the advancement of knowledge and application about space biotechnology [2]. However, SMG was more applicable and feasible to most researchers due to much lower cost, without any further need of spaceflight and extreme sensitive instrumentations, which definitely make the experiments out of range for future projects. Therefore a series of ground-based suspension culture bioreactors which could model various aspects of spaceflight were invented [1]. One such bioreactor named as high-aspect-ratio vessel (HARV, Synthecon) [3] was used in this study. The HARV employed for cell suspension culture and tissue growth permits cell growth in suspension and minimizes the fluid shear levels encountered by cells [1], [4]. When the cell culture vessel rotates, microbial cells do not settle down but revolve around a horizontal axis and continuously fall through the fluid at 1×g terminal velocity condition, which lead to 1∼2×10^−2^ gravity environment [5]. Exchange of dissolved gas has been achieved through a permeable membrane at the back of this vessel.

There were several studies that had demonstrated that microbial cellular processes, such as cell growth, gene expression [6], metabolism [7], and secondary-metabolites production [8], [9] changed when microbial cells were cultured under microgravity or SMG conditions compared with NG. In fact, most of these studies concerning about the effects of microgravity or SMG on the functions of prokaryotic and eukaryotic cells remained focus on the safety and health of astronauts during a long spaceflight time [10], especially supported by NASA. However, we are interested in application of simulated microgravity techniques in biochemical engineering such as recombinant protein production by microbial host and the mechanisms that microbes sense and respond to the environment of SMG.

β-glucuronidase is an important enzyme from *Penicillium purpurogenum* Stoll (**CGMCC 3. 3708**) that can directly hydrolyze glycyrrhizin (GL) to glycyrrhetinic acid monoglucuronide (GAMG) [11]. GAMG is useful in clinical treatment of many inflammatory diseases and much safer, more effective and absorbable than GL [12]. In this study *P. pastoris* is utilized as a host for production of PGUS gene (GenBank Accession **No. EU095019**) because its expression system presents many advantages [13]. Besides its high-level expression of recombinant proteins, *P. pastoris* is also well-known for its strong but tightly regulated alcohol oxidase-1 (AOX1) gene promoter [14]. We previously transformed PGUS gene using the vector pPIC9K into *P. pastoris* GS115 cells and reported that the recombinant *P. pastoris* grew faster under SMG than NG during methanol induction phase and the efficiencies of recombinant PGUS production and secretion were enhanced under SMG as compared with NG control [15]. We hypothesized that the important reason that caused better growth and enhancement of PGUS expression was due to changed transcriptional levels of some key genes, namely SMG condition possibly could regulate molecular responses of the microbial cells. Hence, we examined the transcriptome profiling of the recombinant *P. pastoris* by using NGS as it provides cost-effective, rapid and highly parallel sequencing of large numbers of DNA fragments from complex samples or transcriptomes for gene expression profiling and functional genomics research [16]. Thus we are able to identify transcriptional factors and signaling pathways involved in this special response by figuring out the regulatory motifs of transcriptional profiling of genes, such as, the genes functioned as carbohydrate metabolism, protein synthesis, protein transportation or secretion.

## Materials and Methods

### Strain and Plasmid

The recombinant strain *Pichia pastoris* GS115 used in this study was constructed and preserved in our previous research work [15]. Expression of *pgus* gene was under the control of alcohol oxidase-1 gene promoter for the production of recombinant protein. Standard procedure was adopted for the recombinant *pgus* gene manipulations [17]. The recombinant *P. pastoris* clones with the highest resistance to G418 were used for HARV cultivation.

### Growth conditions

A single colony of recombinant *P. pastoris* clone isolated from the YPD (1% yeast extract, 2% peptone, 2% glucose, and 2% agar) plate was used to inoculate 200 ml of BMGY medium (1% yeast extract, 2% peptone, 100 mmol l^−1^ phosphate buffer saline, pH 6.0, 1.34% YNB, 1.61 µmol l^−1^ biotin, 0.004% histidine, 1% glycerol) in a 500 ml shaker flask and incubated at 30°C and 220 rpm for 8 h to OD_600_ = 2–3. The cells were then harvested by centrifugation at 8000 rpm for 10 min, washed twice with phosphate buffer and resuspended in 100 ml BMMY medium (1% methanol instead of glycerol as the sole carbon source). Then aliquots of the cultures were loaded into two HARV vessels used in this work that were completely filled with 50 mL BMMY culture medium for SMG and NG cultures, respectively. All bubbles were removed to reduce shear using sterile disposable syringe. Methanol was added after every 24 h to a final concentration of 1%. We performed continuous dilutions after every 8 h of induction to guarantee all the cells maintaining in the mid-exponential growth phase by monitoring the optical density. After four repeated processes (16 generations), the culture medium was sampled and centrifuged at 8000 rpm for 15 min at 4°C, then pellets were washed, frozen and stored at −80°C for further RNA isolation. To obtain the cells in stationary phase, four repeated 16 h of the same induction processes (32 generations) were carried out.

### RNA isolation and Samples preparation

Total RNA was isolated using RNA Purification Kit (Amresco Inc, Cochran Road, Solon, USA) according to the manufacturer's instructions. RNA pellets were washed with cold 70% ethanol, air dried, and then resuspended in 30 µL distilled water pretreated with diethylpyrocarbonate (DEPC). Then the extracted RNA were incubated for 60 min at 42°C with 2 µL DNase I (5 U/µL) and 0.5 µL RNase Inhibitor (40 U/µL) (Takara, Dalian, China) to remove residual genomic DNA. After that phenol-chloroform-isopentanol was utilized to dissolve and remove DNase I and RNase Inhibitor. 28 s/18 s rRNA band intensity was determined using Agilent 2100 (Agilent Technologies, US) and the readings of extracted RNA samples between 1.5 and 2 were selected for library construction and sequencing.

### Library preparation and NGS sequencing

We performed the direct sequencing on the Illumina platform with the modified protocol [18]. 6 µg total RNA from exponential and stationary growth phases under SMG and NG conditions captured by magnetic oligo (dT) beads was used for cDNA synthesis. Double stranded bead-bound cDNA was digested with the anchoring enzyme NlaIII. Then the 3′-cDNA fragments were linked to the adapter 1 that included the recognition site of enzyme NlaIII (5′-CATG-3′). Tag fragments with adapter 1 were isolated from the recognition site. After that the adapter 2 (5′-linker, containing a recognition site of MmeI) was added to the site of MmeI digestion. Subsequently all the adapter-ligated cDNA tags were amplified. Each library was then sequenced on an individual lane on an Illumina Genome Analyzer II for 36 cycles. One sample from each time point under SMG and NG was sequenced for a second time with 32 cycles.

### Data analysis and hierarchical clustering

The magnitude of gene transcriptional profiling and abundance of a particular transcript relative to controls in a certain sample are the most desired information. We can obtain the fold change of transcriptional level of a single gene under SMG compared with NG condition using NGS assay, while hierarchical clustering map can give more intuitive result. Hierarchical clustering of the NGS data was performed as previously described [19] using the software Cluster 3.0 (http://bonsai.hgc.jp/~mdehoon/software/cluster). The samples of RNA were obtained from exponential or stationary phases under SMG and NG conditions, respectively. The NGS data of four categories that each comprised of 20 genes related to metabolism and protein synthesis or transportation were clustered. The data from each experiment was weighted and estimated by Cluster 3.0 according to the similarity of expression profiling of each gene to others. The results of cluster were displayed using the software Java TreeView (http://jtreeview.sourceforge.net).

### RT-qPCR analysis

Total RNA (about 0.05 µg) was isolated from *P. pastoris* cultured under SMG and NG conditions and reverse transcribed using a two-step strategy described previously [20] with reverse transcriptase PCR kit (Fermentas). The housekeeping genes (GAPDH) were selected for normalizing expression of the samples. All real-time PCR reactions were performed on the M×3000P (Agilent Technologies, US) with fluorescence signal detection (SYBR Green) after each amplification cycle. Each PCR reaction was performed in a 25.0 µl reaction mixture containing 12.5 µl of 2×SYBR Premix Ex Taq (TaKaRa), 2.0 µl of properly diluted cDNA from 20–30 ng/ml of cDNA for all genes used for RT-qPCR, 0.5 µl of 50×ROX reference dye II (for error correction between wells), 0.5 µl of each primer at 10 µM and 9 µl of sterile distilled water. The negative controls (without cDNA) for each primer set were included in each cycle. The thermal cycling conditions included initial denaturation at 95°C for 30 s, followed by 40 cycles each of denaturation (5 s at 95°C), annealing (20 s at 64°C) and extension (30 s at 72°C with a single fluorescence measurement), and a melt curve program with continuous fluorescence measurement. The data from the real-time PCR was converted to 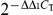 (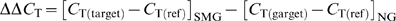) that represents fold change, where *C*_T_ represents the threshold cycle [21], [22]. All the values were determined through triplicate experiments for subsequent analysis. Statistical significance was considered significant at *P*<0.05.

## Results

### Library composition and sequencing of the RNA transcriptomes

The RNA transcriptomes of *P. pastoris* GS115 cultured under SMG and NG conditions were analyzed by NGS using Solexa Genome Analyzer. Library composition was achieved by mapping all short reads longer than 12 nt. About 80% of the sequences were available to map and the comparative extreme long reads, for example above 40 nt, were less than 3%. To investigate the reproducibility of the analysis, two samples derived from either exponential or stationary phase under SMG and NG conditions were sequenced in duplicate (**Table 1**). The low quality tags were filtered and a total of 3716288, 3262032, 3149116, and 5077915 clean tags were obtained. 14514, 10938, 15143, and 10366 distinct tags that matched to unique genes were 25.75%, 24.55%, 17.55%, and 20.95% of total tags, which determined each sequence annotation and expression profiling. In addition, there were over 65% and 50% of the distinct tags available between 2–10 copies, about 15% and 25% of the distinct tags between 11–50 copies, and less than 10% of the tags had more than 51 copies derived from SMG and NG conditions, respectively. Sequencing saturation analysis that was used for estimating the transcriptome coverage for the four data sets was carried out subsequently (**Fig. S1**). It can be concluded that 60%–80% of all the genes have been identified when the number of the sequencing tags was saturated (detected tags reached to 7–10×10^5^).

**Table 1:** Sequencing quality evaluation of the cDNA samples of *P. pastoris* GS115 cultured under SMG and NG conditions.

	Tags sequenced	Stationary (SMG)	Stationary (NG)	Exponential (SMG)	Exponential (NG)
**Number of total tags**	Total tags	3748512	3932994	3748455	5200265
	Distinct tags	87368	61891	87367	82681
**Number of clean tags**	Total tags	3716288	3262032	3149116	5077915
	Distinct tags	76356	70016	106337	85159
**Number of tags matched to gene**	Total tags	568040	800087	568043	450031
	Distribution of total tags	15.28%	24.53%	15.16%	10.06%
	Distinct tags	12058	18082	16894	10998
	Distribution of distinct tags	12.22%	18.65%	15.95%	10.04%
**Number of tags matched to unique gene**	Total tags	1986486	1446734	1987115	659187
	Distribution of total tags	53.45%	44.36%	52.32%	12.98%
	Distinct tags	14514	10938	15143	10366
	Distribution of distinct tags	25.75%	24.55%	17.55%	20.95%
**Number of unknown tags**	Total tags	648151	571776	665218	731177
	Distribution of total tags	17.44%	17.53%	17.76%	22.76%
	Distinct tags	30512	23829	47579	25104
	Distribution of distinct tags	54.14%	59.78%	55.14%	55.55%

### Expression profiling of the four categories of genes

We considered the important reason that led to enhancement of the PGUS expression is probably due to alteration of transcriptional levels of certain kinds of genes which have influences in the recombinant protein expression and transportation. Four categories of genes identified according to Gene Ontology (GO), such as methanol utilization, protein chaperone, RNA polymerase and protein secretion or transportation, were chosen to be analyzed from the data of transcriptome profiling using NGS. The abundance of the filtered genes in the data set has been examined according to the number of transcripts per million (TPM) of distinct clean tags from the NGS results. In order to avoid the gene expression changes caused by the response to growth phases, we performed expression analysis of *P. pastoris* GS115 cultured under SMG and NG conditions at exponential and stationary phases. The false discovery rates (FDR)<0.001 and the value of |log2ratio|≧1 were used as determining the statistical significance of genes expression. Among 4120 normalized and filtered genes, 2705 genes were changed when the recombinant *P. pastoris* responded to the environment of SMG.

**Figure 1:**
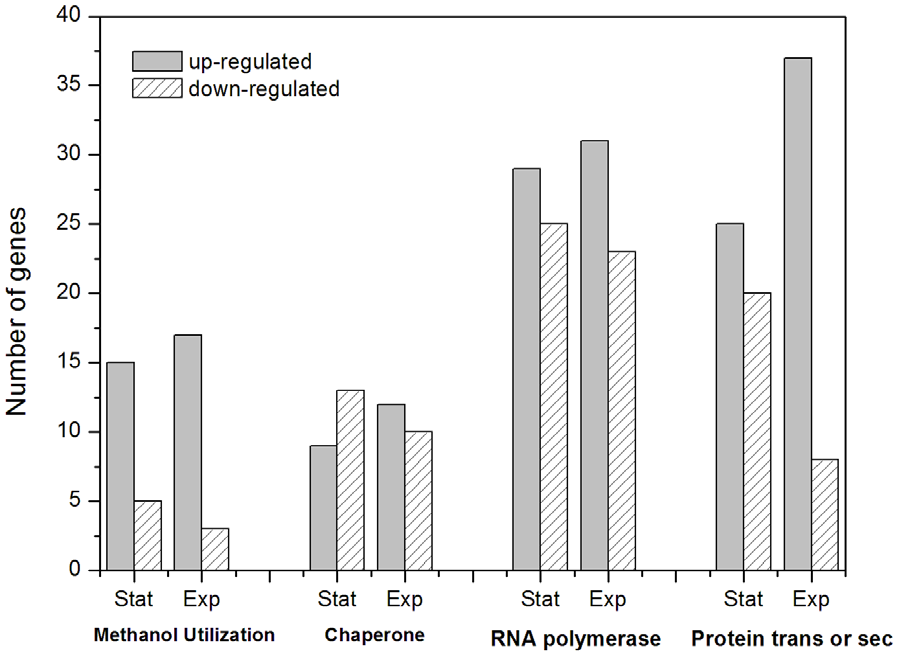
141 significant genes classified using GO resource. These genes functioned as methanol metabolism, chaperone, RNA polymerase, and protein transportation or secretion in *P. pastoris* GS115 differentially responded to SMG condition.

141 genes that function as methanol metabolism, chaperone, RNA polymerase, and protein transportation or secretion classified using GO resource had significant different responses under SMG condition compared with NG control in HARV (**Table 2** **and Table S1, S2**). In these four categories of target genes, 78 were up-regulated and 63 were down-regulated in stationary phase, while 97 up-regulated and 44 down-regulated in exponential phase. The transcriptional level changed notably between genes in these four categories; for example, the gene that functions as essential protein possibly involved in secretion (*PAS_chr3_0292*) was up-regulated 8.3 folds in exponential phase and this level was higher than any other genes while the lowest down-regulated gene (*PAS_chr1-1_0237*) identified as protein chaperone was 6.1 folds. **Figure 1** shows the numbers of significant genes in each category. The transcriptional profiling of each category was similar in the two growth phases. It is interesting to find that the numbers of up-regulated genes related to methanol metabolism (85% of 20 genes) and protein transportation or secretion (82.2% of 45 genes) were about 5 times more than those of down-regulated genes in exponential phase. However, the rest of other two categories had almost no significant differences in the two phases. Furthermore the quantity and fold change of up-regulated genes in exponential phase of each category were higher than that of stationary phase especially the categories of methanol metabolism and protein transportation or secretion.

**Table 2:** Expression patterns of chaperone and protein transportation or secretion genes among the four categories related to protein expression studied in this work during growth under SMG compared with NG control in HARV.

Gene	Gene ID	Fold Change of expression levels^a^				Functional Description
		Stationary Phase		Exponential Phase		
		Up	Down	Up	Down	
**Protein Chaperone**						
PAS_chr3_0480	8199608	3.5		4.1		Putative chaperone
PAS_chr2-1_0323	8199011	1.5		1.4		Essential Hsp90p co-chaperone
PAS_chr2-1_0421	8198889	1.4		3.2		Protein chaperone or co-chaperone in ER
PAS_chr2-1_0140	8198455		1.3		2.2	Chaperone involved in protein folding
PAS_chr2-2_0066	8199171	2.2		2.0		Protein chaperone regulator
PAS_chr2-2_0092	8199196	2.1		1.8		Putative chaperone
PAS_chr4_0051	8201142	1.0		1.8		Hsp40p co-chaperone
PAS_chr2-2_0323	8198223	1.1		1.7		Hsp70 Ssc1p co-chaperone
PAS_chr1-4_0072	8197289	3.7		1.5		Co-chaperone and ATPase activator
PAS_chr2-1_0518	8198440		1.6		1.2	Hsp90p co-chaperone
PAS_chr3_0731	8200426	1.5		1.1		Ribosome-associated molecular chaperone
PAS_chr1-4_0130	8197345		1.1	1.5		Heat shock protein Hsp90
PAS_chr1-3_0063	8197373		1.5	2.0		ER chaperone
PAS_chr1-3_0116	8197585		1.4		1.1	ER packaging chaperone
PAS_chr2-2_0151	8198728		1.4		1.5	Type II Hsp40p co-chaperone
PAS_chr2-2_0015	8198974		1.2		1.4	Putative chaperone DnaJ
PAS_chr1-1_0237	8196739		4.9		6.1	Nucleotide exchange factor of the chaperon Kar2p
PAS_chr1-4_0519	8197103		1.9		2.5	Co-chaperone that stimulates the ATPase Ssa1p
PAS_chr1-3_0137	8197605		1.7		1.9	Molecular chaperone
PAS_chr3_0571	8199935		1.3		2.0	Subunit of the chaperonin Cct ring complex
PAS_chr4_0290	8200732		1.4	2.2		Vacuolar transporter chaperon
PAS_chr1-3_0102	8197412		1.2		1.6	Chaperon in ER
**Protein Transportation or Secretion**						
PAS_chr3_0292	8200207		3.0	8.3		Essential protein possibly involved in secretion
PAS_chr2-1_0342	8199030	2.7		4.2		Secretion promoter
PAS_chr4_0868	8201266	1.3		6.7		ER to Golgi transporter
PAS_chr2-1_0484	8198407	1.5		2.5		Putative ER to Golgi transporter
PAS_chr1-1_0187	8197921	1.2		2.5		Dynein-related ATPase
PAS_chr2-1_0744	8198362		1.9		1.6	Microtubule motor protein
PAS_chr4_0900	8201304	1.9		6.8		Kinesin-like protein
PAS_chr4_0618	8201327	1.8		3.2		Type I myosin
PAS_chr1-4_0271	8196942	1.9		5.9		MAKK in protein kinase C signaling pathway
PAS_FragD_0005	8200509	1.7		3.7		Vacuolar protein sorting factor
PAS_chr3_0143	8199422	2.5		6.3		Rab GTPase essential for exocytosis
PAS_chr2-1_0074	8198470	1.6			2.0	Protein exocytosis regulator
PAS_chr2-1_0056	8198175		1.5		1.2	Palmitoyltransferase that acts on the SNAREs
PAS_chr1-3_0202	8196437	1.3		2.2		Essential subunit of Sec61 complex
PAS_chr2-2_0210	8198618		1.8	3.4		β-subunit of the Sec61p ER translocation complex
PAS_chr1-4_0294	8197744		1.6	1.1		Secretory vesicles locator
PAS_chr1-4_0231	8196905		2.8	2.2		Essential component of the COPII coat of secretory pathway vesicles
PAS_chr4_0165	8201075	2.9		3.4		GTPase involved in the protein secretory pathway
PAS_chr3_0347	8199480	6.6		6.7		Exocytosis regulator
PAS_chr4_0078	8201165	2.9		2.5		Essential protein involved in splicesome assembly and exocytosis
PAS_chr1-4_0452	8197040	1.7		1.3		Essential subunit of exocyst complex
PAS_chr4_0695	8200591	1.6		3.0		Essential subunit of exocyst complex
PAS_chr4_0134	8201046		1.1	2.1		Exocytosis regulator
PAS_chr1-4_0066	8197283		1.2	1.8		Effector of Sec4p to form complex with Sec4p and t-SNARE
PAS_chr4_0704	8200600		1.1		2.4	A component of autophagosomes and Cvt vesicles
PAS_chr4_0098	8201184		1.9		2.3	Subunit of elongator complex
PAS_chr3_0974	8199721		2.0		2.1	ER-Golgi protein transport
PAS_chr1-4_0629	8197837		2.7	1.7		Subunit of the Ssh1 translocon complex
PAS_chr3_0342	8199475		1.5	1.6		ATPase required for the release of Sec17p
PAS_chr4_0284	8200501		1.8	2.0		Cytoplasmic thioredoxin isoenzyme
PAS_chr3_1107	8199694		1.9	1.8		Protein required for fusion of cvt-vesicles
PAS_chr2-1_0199	8198581		1.6		1.8	Putative protein transport
PAS_chr3_0042	8199328	1.4		2.1		Protein kinase involved in vacuolar protein sorting
PAS_chr4_0062	8201150	1.2		2.8		Vacuolar membrane protein
PAS_chr1-4_0528	8197112	1.2		2.1		GTPase of the Ypt/Rab family
PAS_chr4_0395	8200679	2.1		1.9		Essential subunit of Sec63 complex
PAS_chr4_0391	8200675	1.8		2.0		Component of the translocase of outer membrane complex
PAS_chr2-2_0316	8198216	2.1		2.2		Golgi to plasma membrane protein transport
PAS_chr2-1_0572	8198010	1.7		1.9		Protein transport regulator
PAS_chr1-4_0555	8197686	1.5		1.2		Adapter protein for Cvt pathway
PAS_chr2-1_0625	8198939	1.5		2.0		Type I transmembrane sorting receptor
PAS_chr3_0586	8199790		1.6	2.8		Rab escort protein
PAS_chr2-1_0644	8199258		1.6	1.4		Protein required for vesicular transport between ER and Golgi
PAS_chr2-1_0380	8199068		1.4	2.5		Protein involved in nuclear export of the large ribosomal subunit
PAS_chr2-1_0641	8199255		2.1		1.2	Protein required for vesicle formation in autophagy

a Fold change of expression levels = log_2_ Ratio(SMG/NG).

### Hierarchical clustering analysis

**Figure 2:**
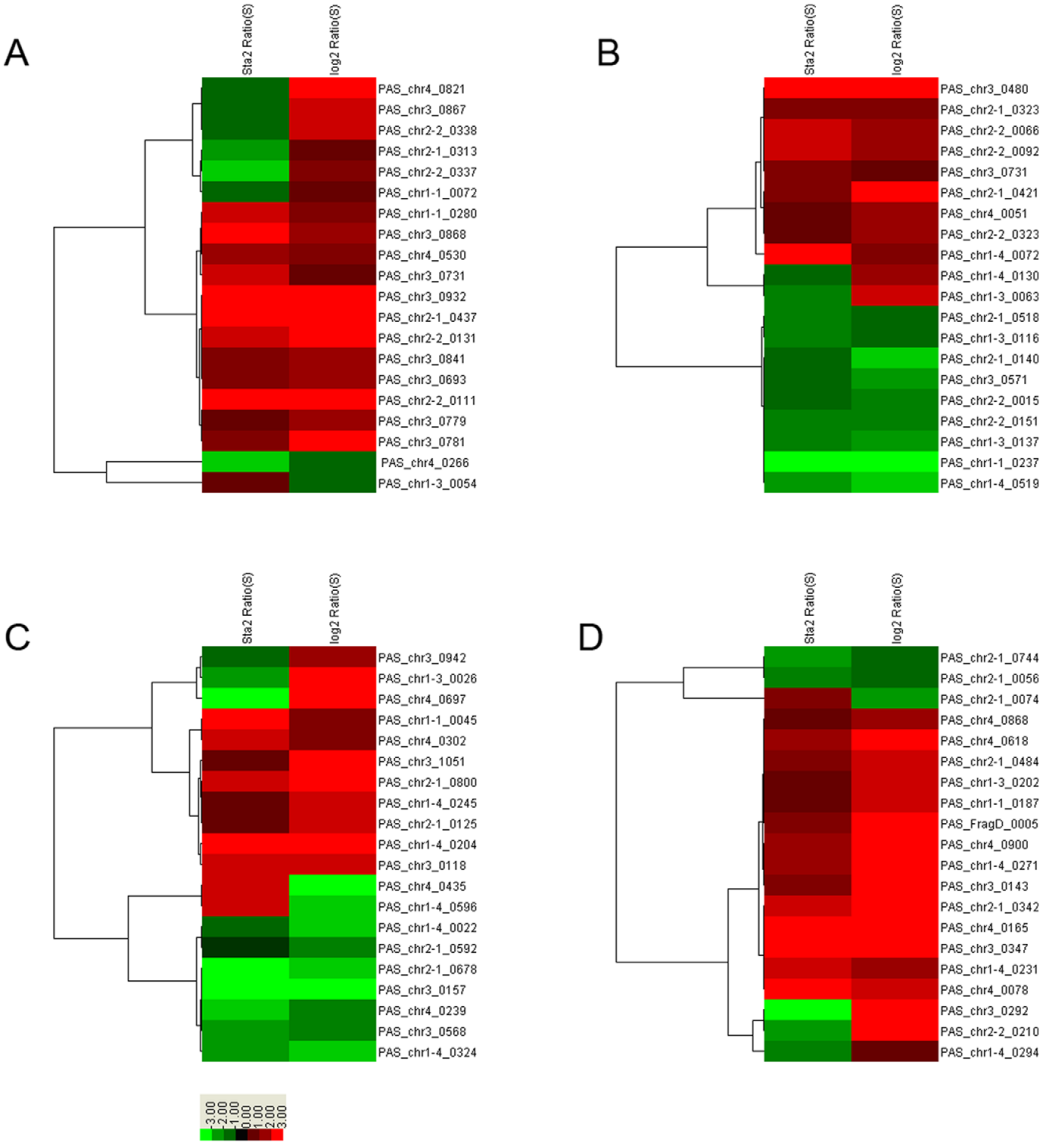
Hierarchical cluster analysis of the expression profile of four functional categories of genes under SMG and NG. (A) methanol utilization; (B) protein chaperone; (C) RNA polymerase and (D) protein secretion or transportation. The bottom color bar represents the expression level of each gene, which red and green denote to up-regulated and down-regulated, respectively. Log2 Ratio and sta2 Ratio represent the cDNA samples were achieved from exponential or stationary phases, respectively.

80 most differentially expressed genes with at least 1.5 fold changes in four categories (20 genes per category) were clustered by average linkage using Cluster 3.0. A two-dimensional hierarchical cluster heat map showed the overall transcriptional response of the *P. pastoris* GS115 cells cultured under SMG and NG environments in HARVs (**Fig. 2**). Gene ID was given to the right of the colored image and the corresponding source of the growth phases were shown at the top of the bands. The red and green bands that denoted to up-regulated and down-regulated, respectively represent fold change of expression level of each gene under SMG compared with NG. Similar as **Table 2** and **Table S1, S2**, most genes related to methanol metabolism and protein secretion were up-regulated in both growth phases. And the degree of changes of these two categories was significantly higher than genes related to protein chaperone and RNA polymerase.

### Real time PCR analysis

**Figure 3:**
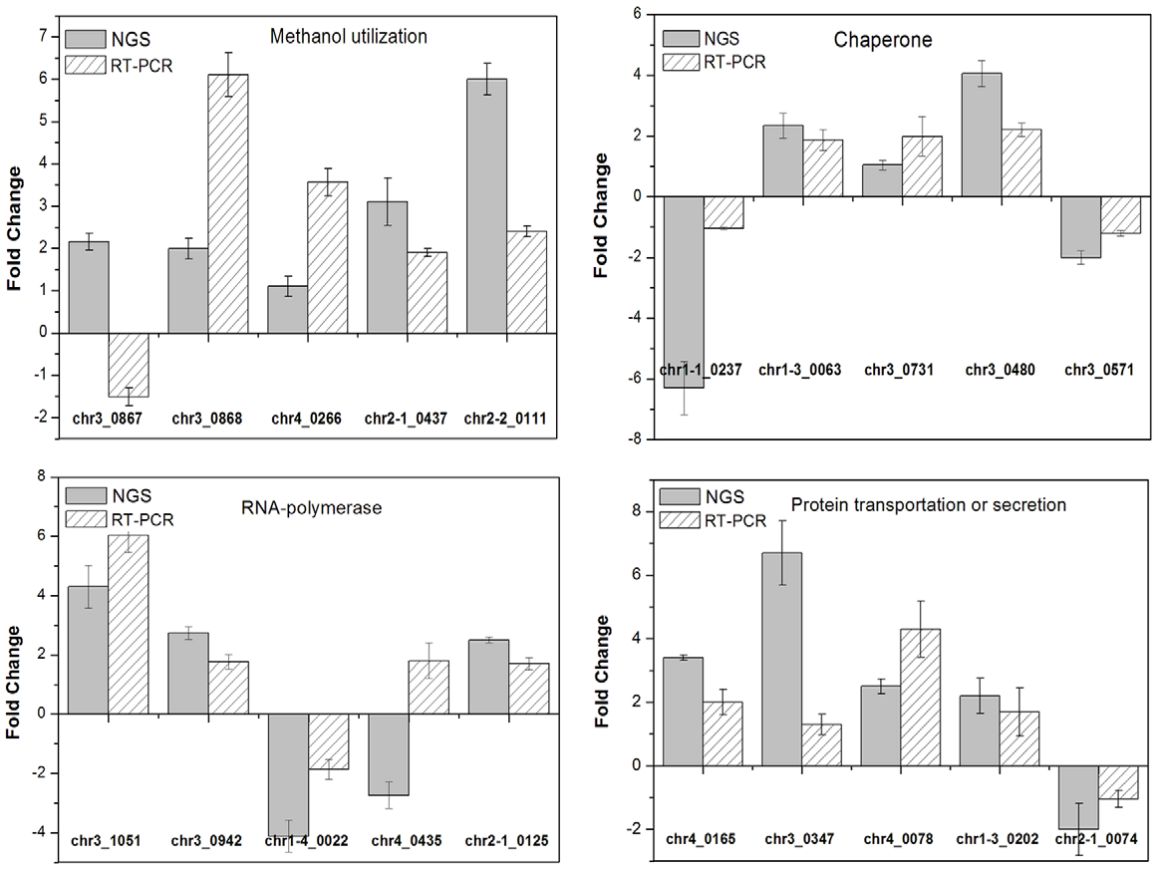
Comparison results of NGS and real time RT-PCR. Expression profiling of 20 genes chosen from four different functional categories according to the cluster analysis were examined using real time RT-PCR. The cDNA samples used in this comparison were extracted from exponential phase.

We compared the NGS expression levels with real time quantitative PCR for further verification of the NGS results. Five genes in each category were selected at random for quantitative real time PCR analysis (**Fig. 3**). Although minor variation of transcriptional levels appeared between the two analyses, most of the tested 20 genes (18/20) matched well in the expression fold change and directions (up- and down-regulated). However two genes (*PAS_chr3_0867* and *PAS_chr4_0435*) show different results between NGS and real time PCR data sets.

## Discussion

Simulated microgravity that had been considered as an extreme and special environment presents many novel aspects to study microorganisms. And studies concerning the influence of SMG on microbial cells are receiving much attention. The changed physiology and metabolism of microbes, such as shortened lag phase, increase in growth rate and higher final cell counts, would happen in the low shear fluid environment of SMG [23], [24]. We previously reported that growth of *P. pastoris* GS115 accelerated and the efficiencies of the recombinant PGUS production and secretion were enhanced under SMG compared with NG control. It is considered that the important reason that causes faster growth and enhancement of PGUS expression may be related to altered genes transcription. However the molecular mechanisms by which the culture conditions of SMG in the HARV modulated gene expression in *P. pastoris* remained unknown.

In order to find the mechanism of enhanced PGUS production under SMG environment, transcriptional levels of the recombinant *P. pastoris* GS115 cultured in exponential and stationary phases were determined using NGS on the Illumina platform. All the distinct tag-mapped genes were identified in each library and the tendency of sequenced tags indicated that saturated numbers of matched genes is obtained. Although part of the filtered tags can not match the correct sequence of *P. pastoris* GS115 genome, these “mismatched” tags have no influence on the construction of each library since they probably represent the novel genes to be identified. There are still a large proportion of unique distinct tags enough for library construction. For the changed growth and recombinant protein expression, we summarized the transcriptional profiling of four categories of genes that were related to metabolism and protein expression such as methanol utilization, protein chaperone, RNA polymerase and protein secretion or transportation. Then we applied the cluster to four sets of NGS data including significant 80 genes (20 genes of each category) and the hierarchical cluster map gave more intuitive comparison.

For the four categories of genes, the quantity and fold change of up-regulated genes in exponential phase of each category were higher than stationary phase, because the whole process of protein synthesis, folding, transportation or secretion is more active at exponential phase of the cells. Among the significant changed 141 genes, most genes related to methanol metabolism and protein transportation or secretion had been up-regulated in both growth phases. *P. pastoris* GS115 cells were cultured under the same conditions of the first stage in which glycerol was used as sole carbon source and there was only one difference (SMG and NG) in the second stage of induction in which methanol was used as the sole carbon resource instead of glycerol. The up-regulated methanol metabolism genes directly led to more efficient utilization of the only carbon source and ultimately more final cell counts were achieved. The alcohol oxidase gene (*PAS_chr4_0821*) that was the major source of methanol-oxidizing activity in methanol-grown *P. pastoris* had been up-regulated 3.6 folds in exponential phase under SMG. In fact, alcohol oxidase gene has been proved to be a strong but tightly regulated promoter [14]. Thus the significant change of this gene would cause faster oxygen and methanol uptake, which promoted transcription of the recombinant gene. As well as alcohol oxidase gene, several key genes coding for the respective enzymes involved in methanol metabolism pathway in *P. pastoris*, for example, glycerol-3-phosphate dehydrogenase (*PAS_chr2-2_0111*), NAD(+)-dependent formate dehydrogenase (*PAS_chr3_0932*) and catalase A (*PAS_chr2-2_0131*), were highly up-regulated for 6.0, 4.0 and 2.4 folds in exponential phase, respectively. Furthermore, the transcriptional levels of these genes were all up-regulated for 2.8 folds at least in stationary phase. Due to the optimized laminar flow condition and the minimized mechanical stresses of SMG [5], the results also indicated that the unique environment of SMG could significantly strengthen the transcriptional responses of *P. pastoris* in methanol utilization and metabolism at the induction stage.

Interestingly, genes functioned as protein transportation or secretion were found also highly up-regulated. And even transcriptional levels of the down-regulated genes were comparative lower than other categories. It is likely that these up-regulated genes also contribute to enhance production of the recombinant protein under SMG environment. Demain et al had reported that in most cases secretion of secondary metabolites was inhibited under SMG [8]. The environment around the cell cultured under SMG turns to be a special zone with less nutrition but high concentration of metabolites [23], which probably cause reduced secretion of secondary metabolites. However it was concluded that the process of recombinant protein production by microbial cells under SMG was different from the secondary metabolites. We considered that SMG probably was a condition facilitating secretion of protein or even other biological macromolecules, especially extracellular secretion because of much higher up-regulated genes related to exocytosis or extracelluar secretion (such as *PAS_chr3_0292, PAS_chr3_0347, PAS_chr4_0078 and PAS_chr2-2_0210*). Furthermore, the genes of protein secretion were higher up-regulated than genes of protein transportation because genes of protein secretion might be more affected by SMG, or otherwise the method of GO classification was not very applicable in protein transportation or secretion. In addition, most of the tested 20 genes matched well in the two methods (NGS and real time RT-PCR) in spite of some difference in transcriptional profiling. The disparity in the expression levels of these genes using NGS and quantitative PCR analysis might be attributed to the differences in methanol induction process.

It seems that SMG can be regarded as a special kind of environment signal or stress that is quickly sensed by cells. The cells have to respond to the stress and ultimately adapt themselves to this environment, and then physiological changes of the recombinant *P. pastoris* could be observed. We concluded that enhanced production of the recombinant protein under SMG had close relation with the intensified transcriptional levels of two kind of key genes; in fact, our previous results and hypothesis had also been confirmed [15]. And we will proceed to further investigation of the mechanism through which microbial cells sense the reduced gravity conditions and also how they convert these mechanical signals into molecular and biochemical responses. This can give rise to the advanced knowledge about the pathway and mechanism of biological process under SMG.

## Supporting Information

Figure S1 **Sequencing saturation analysis of the gene tags libraries of** ***P. pastoris*** **GS115.** (A) stationary phase and (B) exponential phase under SMG (S) and NG (N) environments, respectively.
(RAR)

Table S1 **Expression patterns of 20 significant genes related to methanol utilization under SMG compared with NG.**
(DOC)

Table S2 **Expression patterns of 54 significant genes related to RNA polymerase under SMG compared with NG.**
(DOC)
