# Supplementary material for: Transcriptional Profiling of Protein Expression Related Genes of Pichia pastoris under Simulated Microgravity
Source: PLoS One. 2011 Nov 2;6(11):e26613. doi: 10.1371/journal.pone.0026613 (PMC3206813; doi:10.1371/journal.pone.0026613)
Supplement: Table S1 — Expression patterns of 20 significant genes related to methanol utilization under SMG compared with NG. (DOC) [file pone.0026613.s002.doc]

**Table S1**

| **Gene** | **Gene ID** | **Fold Change of expression levels ^a^** | | **Functional Description** |
| --- | --- | --- | --- | --- |
|  |  | Stationary Phase | [Logarithmic](http://dict.baidu.com/s?wd=logarithmic) Phase |  |
|  |  | Up Down Up Down | |  |
| **Methanol Utilization** |  |  |  |  |
| PAS_chr2-2_0111 | 8199215 | 4.2 | 6.0 | Glycerol-3-phosphate dehydrogenase |
| PAS_chr4_0821 | 8201223 | 1.4 | 3.6 | Alcohol oxidase |
| PAS_chr3_0867 | 8200354 | 1.8 | 2.1 | Non-essential intracellular esterase |
| PAS_chr2-1_0313 | 8199001 | 1.7 | 1.2 | Bifunctional enzyme |
| PAS_chr3_0932 | 8200284 | 3.6 | 4.0 | NAD(+)-dependent formate dehydrogenase |
| PAS_chr2-2_0131 | 8198267 | 2.4 | 2.8 | Catalase A |
| PAS_chr3_0841 | 8200330 | 1.3 | 1.8 | Dihydroxyacetone kinase |
| PAS_chr3_0868 | 8199670 | 2.9 | 2.0 | Fructose-1,6-bisphosphatase |
| PAS_chr1-1_0280 | 8197931 | 2.4 | 1.5 | Xylulokinase |
| PAS_chr4_0266 | 8200935 | 2.3 | 1.3 | 6-phosphofructo-2-kinase/fructose-2,6-bisphosphatase |
| PAS_chr3_0731 | 8200426 | 2.2 | 1.1 | Cytoplasmic ATPase |
| PAS_chr4_0530 | 8201008 | 2.1 | 1.6 | Cytosolic aspartate aminotransferase |
| PAS_chr3_0781 | 8200474 | 1.5 | 3.0 | Glycogen synthase |
| PAS_chr1-3_0054 | 8196821 | 1.2 | 1.1 | Phosphatase subunit |
| PAS_chr2-1_0437 | 8198905 | 2.8 | 3.1 | Glyceraldehyde-3-phosphate dehydrogenase |
| PAS_chr2-2_0337 | 8198237 | 2.1 | 1.5 | Transaldolase |
| PAS_chr1-1_0072 | 8197200 | 1.3 | 1.2 | 1,6-bisphosphate aldolase |
| PAS_chr2-2_0338 | 8198238 | 1.3 | 2.5 | Transaldolase |
| PAS_chr3_0693 | 8200393 | 1.4 | 1.7 | Tetrameric phosphoglycerate mutase |
| PAS_chr3_0779 | 8199657 | 1.1 | 1.9 | Ubiquitin-conjugating enzyme |

**Expression patterns of 20 significant genes related to methanol utilization under SMG compared with NG**

^a^ Fold change of expression levels= log_2_ Ratio(SMG/NG)
