# Supplementary material for: Transcriptional Profiling of Protein Expression Related Genes of Pichia pastoris under Simulated Microgravity
Source: PLoS One. 2011 Nov 2;6(11):e26613. doi: 10.1371/journal.pone.0026613 (PMC3206813; doi:10.1371/journal.pone.0026613)
Supplement: Table S2 — Expression patterns of 54 significant genes related to RNA polymerase under SMG compared with NG. (DOC) [file pone.0026613.s003.doc]

**Table S2**

| **Gene** | **Gene ID** | **Fold Change of expression levels ^a^** | | **Functional Description** |
| --- | --- | --- | --- | --- |
|  |  | Stationary Phase | [Logarithmic](http://dict.baidu.com/s?wd=logarithmic) Phase |  |
|  |  | Up Down Up Down | |  |
| **RNA polymerase** |  |  |  |  |
| PAS_chr3_0942 | 8200293 | 1.2 | 1.7 | RNA polymerase III subunit C11 |
| PAS_chr4_0435 | 8200717 | 2.6 | 2.7 | Subunit of the RNA polymerase II mediator complex |
| PAS_chr3_1051 | 8200012 | 1.1 | 4.3 | RNA polymerase III subunit C17 |
| PAS_chr1-4_0245 | 8196919 | 1.1 | 2.7 | Component of the core form of RNA polymerase transcription factor TFIIH |
| PAS_chr1-1_0045 | 8197562 | 3.0 | 1.45 | Largest of six subunits of the RNA polymerase III transcription initiation factor complex |
| PAS_chr1-3_0026 | 8197959 | 1.9 | 4.8 | Negative regulator of RNA polymerase III |
| PAS_chr2-1_0678 | 8199291 | 2.8 | 2.2 | Protein involved in the transcription of 35S rRNA genes by RNA polymerase |
| PAS_chr2-1_0800 | 8198077 | 2.2 | 4.1 | Subunit of the RNA polymerase II mediator complex |
| PAS_chr1-4_0022 | 8196862 | 1.1 | 2.1 | Mitochondrial RNA polymerase |
| PAS_chr4_0697 | 8201017 | 4.4 | 4.0 | RNA polymerase III subunit C34 |
| PAS_chr1-4_0204 | 8197186 | 2.8 | 3.9 | Component of the holoenzyme form of RNA polymerase transcription factor TFIIH |
| PAS_chr4_0302 | 8200975 | 2.7 | 1.6 | RNA polymerase III subunit C160, part of core enzyme |
| PAS_chr2-1_0592 | 8198029 | 1.2 | 1.6 | Presumed helicase required for RNA polymerase II transcription termination and processing of RNAs |
| PAS_chr4_0239 | 8200908 | 2.4 | 1.7 | Second-largest subunit of RNA polymerase III |
| PAS_chr3_0157 | 8199432 | 3.4 | 2.9 | RNA polymerase subunit ABC27, common to RNA polymerases I, II and III |
| PAS_chr3_0118 | 8199398 | 2.5 | 2.6 | One of six subunit of RNA polymerase III transcription initiation factor complex (TFIIIC) |
| PAS_chr2-1_0125 | 8198521 | 1.0 | 2.5 | RNA polymerase II second largest subunit B150, part of central core |
| PAS_chr1-4_0596 | 8197726 | 2.2 | 2.4 | Subunit of RNA polymerase II-associated Paf1 complex |
| PAS_chr3_0568 | 8199932 | 2.0 | 1.3 | RNA polymerase II largest subunit B220 |
| PAS_chr1-4_0324 | 8199657 | 1.1 | 1.9 | RNA polymerase I subunit A135 |
| PAS_chr2-1_0780 | 8198057 | 1.3 | 8.0 | Transription coactivator, component of the ADA and SAGA transcriptional adaptor/HAT complexes |
| PAS_chr2-2_0135 | 8198713 | 1.1 | 6.2 | General transcription elongation factor TFIIS |
| PAS_chr1-4_0523 | 8197107 | 5.3 | 8.1 | Transcription/RNA-processing factor |
| PAS_chr2-1_0347 | 8199035 | 1.2 | 4.0 | Protein kinase |
| PAS_chr1-1_0095 | 8197912 | 1.4 | 4.2 | Subunit (60 kDa) of TFIID and SAGA complexes |
| PAS_chr3_0051 | 8199337 | 1.0 | 3.9 | Subunit of the SAGA and SAGA-like transcriptional regulatory complexes, interacts with Spt15p to act |
| PAS_chr3_1035 | 8199997 | 1.8 | 3.6 | Component of the Paf1p complex |
| PAS_chr2-1_0723 | 8198341 | 2.2 | 3.3 | Transcriptional activator related to Msn2p |
| PAS_chr1-4_0365 | 8197813 | 2.9 | 3.0 | Subunit of the SAGA transcriptional regulatory complex but not present in SAGA-like complex SLIK/SAL |
| PAS_chr2-1_0588 | 8198025 | 1.6 | 3.0 | Subunit (61/68 kDa) of TFIID and SAGA complexes |
| PAS_chr2-2_0284 | 8198289 | 3.4 | 2.9 | Transcriptional regulator involved in glucose repression of Gal4p-regulated genes |
| PAS_chr1-4_0094 | 8197311 | 2.0 | 2.8 | GTP binding protein |
| PAS_chr2-2_0061 | 8199166 | 1.0 | 2.7 | Essential abundant protein involved in regulation of transcription |
| PAS_chr2-1_0608 | 8199144 | 3.6 | 2.6 | Activating gamma subunit of the AMP-activated Snf1p kinase complex |
| PAS_chr4_0475 | 8201422 | 2.1 | 2.6 | Essential protein involved in transcription regulation |
| PAS_chr4_0475 | 8197637 | 1.2 | 2.4 | Zinc-finger DNA-binding protein |
| PAS_chr3_0590 | 8199794 | 1.4 | 2.5 | Homeodomain-containing transcriptional repressor of PTR2 |
| PAS_FragD_0018 | 8200520 | 1.0 | 2.3 | Catalytic subunit of the SWI/SNF chromatin remodeling complex involved in transcriptional regulation |
| PAS_chr2-1_0468 | 8198391 | 1.1 | 2.1 | Catalytic (alpha) subunit of C-terminal domain kinase I (CTDK-I), which phosphorylates the C-termina |
| PAS_chr2-1_0277 | 8198826 | 1.2 | 1.2 | TFIIA large subunit |
| PAS_chr2-1_0428 | 8198896 | 1.2 | 1.0 | One of two nearly identical (see HTB1) histone H2B subtypes |
| PAS_chr3_0051 | 8199337 | 3.9 | 1.1 | Subunit of the SAGA and SAGA-like transcriptional regulatory complexes, interacts with Spt15p to act |
| PAS_chr3_0267 | 8200205 | 3.2 | 1.4 | TFIID subunit (48 kDa) |
| PAS_chr1-4_0292 | 8297742 | 1.6 | 1.4 | 3-phosphoglycerate kinase |
| PAS_chr1-1_0019 | 8197536 | 3.1 | 1.4 | Component of the TREX complex required for nuclear mRNA export |
| PAS_chr1-1_0095 | 8197912 | 4.3 | 1.5 | Subunit (60 kDa) of TFIID and SAGA complexes |
| PAS_chr4_0490 | 8201426 | 3.2 | 1.5 | Component of the CCR4-NOT transcriptional complex |
| PAS_chr2-1_0142 | 8198457 | 1.5 | 1.6 | Subunit (17 kDa) of TFIID and SAGA complexes, involved in RNA polymerase II transcription initiation |
| PAS_chr4_0102 | 8201188 | 1.2 | 2.8 | Nuclear protein required for transcription of MXR1 |
| PAS_chr2-1_0375 | 8199063 | 1.8 | 2.8 | Component of the RSC chromatin remodeling complex |
| PAS_chr2-2_0246 | 8198653 | 2.6 | 1.8 | Subunit of the HIR complex, a nucleosome assembly complex involved in histone gene transcription |
| PAS_chr2-1_0394 | 8198862 | 1.7 | 1.9 | TFIIIB B-related factor |
| PAS_chr2-1_0492 | 8198415 | 3.6 | 1.9 | Protein whose overexpression suppresses growth defect of mutants lacking protein kinase A activity |
| PAS_chr1-4_0342 | 8197791 | 8.3 | 4.9 | Essential protein with similarity to phosducins, which are G-protein regulators |

**Expression patterns of 54 significant genes related to RNA polymerase in protein expression studied in this work during growth under SMG compared with NG control in HARV.**

^a^ Fold change of expression levels= log_2_ Ratio(SMG/NG)
